# The emerging role of 5-hydroxymethylcytosine in neurodegenerative diseases

**DOI:** 10.3389/fnins.2014.00397

**Published:** 2014-12-05

**Authors:** Sahar Al-Mahdawi, Sara Anjomani Virmouni, Mark A. Pook

**Affiliations:** ^1^Ataxia Research Group, Division of Biosciences, Department of Life Sciences, College of Health and Life Sciences, Brunel UniversityLondon, Uxbridge, UK; ^2^Synthetic Biology Theme, Institute of Environment, Health and Societies, Brunel University LondonUxbridge, UK

**Keywords:** 5-hydroxymethylcytosine, Alzheimer's disease, amyotrophic lateral sclerosis, fragile X-associated tremor/ataxia syndrome, Friedreich ataxia, Huntington's disease, Parkinson's disease

## Abstract

DNA methylation primarily occurs within human cells as a 5-methylcytosine (5mC) modification of the cytosine bases in CpG dinucleotides. 5mC has proven to be an important epigenetic mark that is involved in the control of gene transcription for processes such as development and differentiation. However, recent studies have identified an alternative modification, 5-hydroxymethylcytosine (5hmC), which is formed by oxidation of 5mC by ten-eleven translocation (TET) enzymes. The overall levels of 5hmC in the mammalian genome are approximately 10% of 5mC levels, although higher levels have been detected in tissues of the central nervous system (CNS). The functions of 5hmC are not yet fully known, but evidence suggests that 5hmC may be both an intermediate product during the removal of 5mC by passive or active demethylation processes and also an epigenetic modification in its own right, regulating chromatin or transcriptional factors involved in processes such as neurodevelopment or environmental stress response. This review highlights our current understanding of the role that 5hmC plays in neurodegenerative diseases, including Alzheimer's disease (AD), amyotrophic lateral sclerosis (ALS), fragile X-associated tremor/ataxia syndrome (FXTAS), Friedreich ataxia (FRDA), Huntington's disease (HD), and Parkinson's disease (PD).

## Introduction

Methylation of mammalian DNA occurs by DNA methyltransferase (DNMT) enzymatic conversion of cytosine residues in CpG dinucleotides to 5-methylcytosine (5mC) (Robertson, 2001). CpG sites are clustered together as CpG islands (CGIs), which locate to distinct gene regions, and the DNA methylation profile of a gene has been shown to have a significant impact upon its level of expression (Bird and Wolffe, 1999). Furthermore, aberrant DNA methylation profiles are known to be associated with many different human diseases, including Rett syndrome (Amir et al., 1999) and cancer (Kulis and Esteller, 2010), where there are alterations of global DNA methylation patterns, and Fragile X syndrome (FXS), where there is specific methylation of the CCG repeat mutation in the fragile X mental retardation-1 (*FMR1*) gene (Naumann et al., 2009). Abnormal global or localized DNA methylation patterns have also been associated with certain neurodegenerative diseases (Pook, 2012; Lu et al., 2013).

In 2009, two independent studies simultaneously reported the existence of an alternative modification, 5-hydroxymethylcytosine (5hmC), formed due to oxidation of 5mC by ten-eleven translocation (TET) enzymes, a family of 2-oxoglutarate- and Fe(II)-dependent dioxygenases, consisting of three proteins, TET1, TET2, and TET3 (Kriaucionis and Heintz, 2009; Tahiliani et al., 2009). Highly conserved homologous proteins have also been identified in mouse, designated Tet1, Tet2, and Tet3. Initial studies demonstrated roles for Tet enzymes, converting 5mC to 5hmC, in pluripotency and developmental reprogramming (Ito et al., 2010; Branco et al., 2011; Ficz et al., 2011; Gu et al., 2011; Inoue and Zhang, 2011). Subsequent studies in mouse embryonic stem (ES) cells suggested that Tet enzymes could control DNA methylation both by the conversion of 5mC to 5hmC and by binding to CpG rich regions to prevent DNMT activity (Xu et al., 2011). More recently, outside of developmental reprogramming, TET1 has been shown to act in differentiated cells as a maintenance demethylase that prevents aberrant methylation spreading into unmethylated or hypomethylated CGIs (Jin et al., 2014). The overall levels of 5hmC in the mammalian genome have been reported to be approximately 10% of 5mC levels (Branco et al., 2011), although higher levels have been detected in tissues of the CNS (Globisch et al., 2010). For example, 5hmC is approximately 40% as abundant as 5mC in the DNA of Purkinje cells of the cerebellum (Kriaucionis and Heintz, 2009). Subsequent to the identification of 5hmC, two other modifications of cytosine have been discovered, 5-formylcytosine (5fC) and 5-carbamylcytosine (5caC). These are less abundant than 5hmC and they are recognized as intermediates generated by TET enzyme activity in the 5hmC to cytosine conversion pathway (Ito et al., 2011).

Several research groups have studied the distribution of 5hmC throughout the genome. In brain tissue, 5hmC is particularly enriched within synaptic genes, exhibiting tissue-specific differences at exon-intron boundaries, suggesting a potential role for 5hmC in the differential splicing of these genes (Khare et al., 2012). Conversely, in human and mouse ES cells, 5hmC is enriched at specific gene bodies, promoters and enhancers, particularly at promoters marked with H3K4me3 and H3K27me3 and at enhancers marked with H3K4me1 and H3K27ac, suggesting a role for 5hmC in the epigenetic regulation of transcription (Pastor et al., 2011; Stroud et al., 2011; Szulwach et al., 2011a). Furthermore, enrichment of 5hmC at both gene bodies of actively transcribed genes and promoter regions of Polycomb-repressed developmental regulator genes has provided evidence of a dual role for 5hmC in pluripotent stem cells (Wu et al., 2011). 5hmC is also found in mitochondrial DNA (Iacobazzi et al., 2013), which has implications for disorders of mitochondrial dysfunction, including neurodegenerative diseases. Due to the CNS-selective tissue distribution of 5hmC and its involvement in epigenetic gene regulation during neurodevelopment, several recent investigations have focused on uncovering potential roles for 5hmC in neurodegenerative diseases. Here we summarize the findings of this recent epigenetic-based neurological research.

## The functions of 5hmC

**Figure 1:**
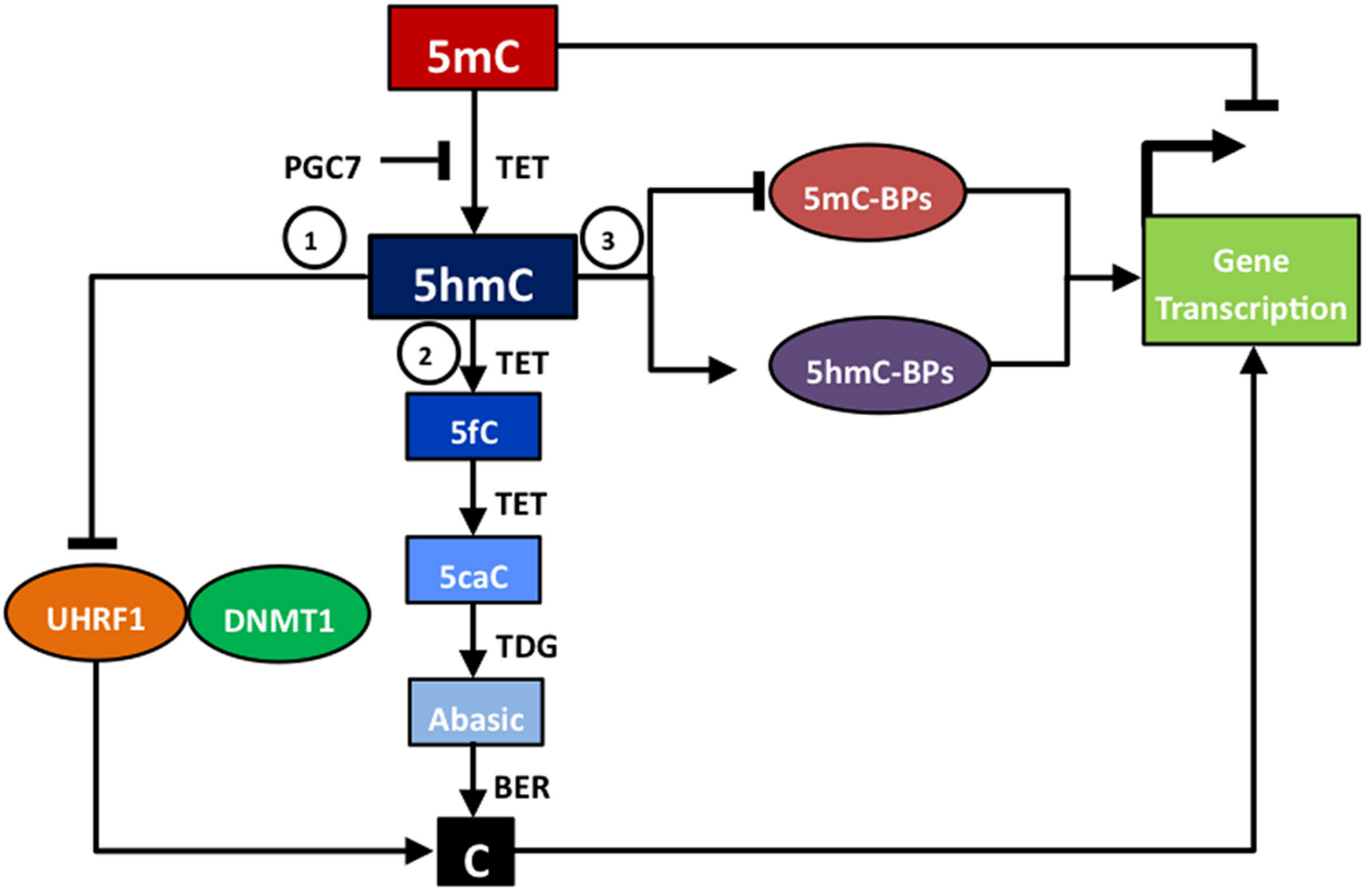
**Functions of 5hmC**. 5hmC has several different functions that impact upon gene transcription: (1) acting as an intermediate in passive DNA demethylation due to poor binding between 5hmC and UHRF1, the partner of DNMT1; (2) acting as an intermediate in TET/TDG/BER-based active DNA methylation; (3) altering the ratio of 5mC-binding proteins (5mC-BPs) to 5hmC-binding proteins (5hmC-BPs) that impair or activate gene transcription.

Although the functions of 5hmC within the cell are not yet fully known, evidence emerging from recent 5hmC protein binding studies supports the existence of multiple roles (Figure 1). Several proteins have now been shown to bind to 5hmC, including UHRF1 (ubiquitin-like, containing PHD, and RING finger domain 1) (Frauer et al., 2011), MBD3 (methyl-CpG binding domain protein 3) (Yildirim et al., 2011), MeCP2 (methyl-CpG binding protein 2) (Mellen et al., 2012), UHRF2 (ubiquitin-like, containing PHD and RING finger domain 2) and a number of other proteins identified by proteomics analysis (Spruijt et al., 2013). These studies suggest that 5hmC is involved, both indirectly and directly, in the dynamic interplay between DNA methylation status and gene transcription.

Firstly, 5hmC acts as an intermediate in both the passive and the active DNA demethylation conversion of 5mC to cytosine, and thus is indirectly involved in regulating gene transcription. Passive DNA demethylation occurs due to poor binding of UHRF1 to 5hmC. Usually UHRF1 and its partner, DNMT1, act together to bind to 5mC and carry out maintenance DNA methylation (Bostick et al., 2007). However, UHRF1 has a reduced binding affinity for 5hmC compared to 5mC (Frauer et al., 2011), and therefore DNMT1 may not be recruited to maintain levels of DNA methylation and hence passive demethylation occurs. Shortly after the discovery that TET enzymes mediated the conversion of 5mC to 5hmC, other studies revealed that TET enzymes could further oxidize 5hmC to 5fC and then 5caC (Ito et al., 2011). This led to the understanding that active DNA demethylation can occur through sequential stages of TET enzyme-mediated oxidation, 5mC to 5hmC to 5fC to 5caC, followed by the action of TDG to form an abasic site, which is then repaired to a cytosine residue by BER activity (Guo et al., 2011; He et al., 2011; Maiti and Drohat, 2011). An alternative active DNA demethylation pathway that involves deamination of 5hmC to a hydroxyuracil (5hmU) intermediate has also been proposed (Cortellino et al., 2011). In addition, there is preliminary *in vitro* evidence that the *de novo* DNA methyltransferases DNMT3A and DNMT3B can act as DNA dehydroxymethylases, which may be able to directly convert 5hmC to cytosine (Chen et al., 2012). Therefore, changes in 5hmC status may simply reflect changes in the biological processes that require DNA demethylation, such as the development of pre-implantation embryos or the reprogramming of primordial germ cells (PGCs) (Kohli and Zhang, 2013). Global DNA demethylation occurs during two stages of embryogenesis: (i) in zygotes where there is preferential DNA demethylation of the parental genome, (ii) in PGCs during the establishment of gender-specific DNA methylation patterns (Inoue and Zhang, 2011). Tet1 is not responsible for global demethylation in PGCs, but has been shown to mediate locus-specific demethylation of a subset of meiotic genes (Yamaguchi et al., 2012) and to have a critical function in the erasure of genomic imprinting (Yamaguchi et al., 2013).

Secondly, 5hmC binds chromatin regulator proteins, which suggests that it is not merely an intermediate in DNA demethylation, but that it can more directly influence the regulation of gene transcription in processes such as neurodevelopment (Szulwach et al., 2011b) or cellular responses to oxidative stress (Chia et al., 2011). For example, 5hmC may modulate the relative binding of methyl-CpG-binding domain proteins, such as MeCP2 and MBD3, to produce a more open chromatin state and activation of gene transcription (Yildirim et al., 2011; Mellen et al., 2012). Furthermore, 5hmC can be associated with, or affected by, particular histone modifications that influence gene transcription. For example, tight correlations of 5hmC localization have been reported with both histone H3K4me2, an epigenetic mark of euchromatin, and H3K27me3, an epigenetic mark of heterochromatin, across a variety of somatic tissues (Haffner et al., 2013; Chen et al., 2014). In addition, recent studies have shown that the conversion of 5mC to 5hmC can be prevented by binding of PGC7 (also known as Dppa3 or Stella) to histone H3K9me2 (Nakamura et al., 2012). Furthermore, as with 5hmC, it is possible that 5fC and 5caC may also have independent functions in the regulation of gene transcription (Raiber et al., 2012).

## 5hmC, neurodevelopment and neurodegenerative diseases

Several studies have suggested a role for 5hmC in the epigenetic regulation of transcription, mediating brain development and functional maintenance of the adult brain. At the outset, comparatively high levels of 5hmC were detected in CNS tissues, which contain predominantly non-proliferating cells (Globisch et al., 2010). Thus, 5hmC was found to be approximately 40% as abundant as 5mC in the DNA of Purkinje cells of the cerebellum (Kriaucionis and Heintz, 2009). In contrast, loss of global 5hmC has been associated with cancer, suggesting that 5hmC cannot be well maintained in highly proliferating cells (Pfeifer et al., 2013). Throughout the stages of mouse neurodevelopment from embryonic to adult brain, 5hmC has been shown to be not merely an intermediate metabolite of DNA demethylation, but a long-lasting but dynamic epigenetic mark that is distinct from 5mC. Thus, while 5mC differentially binds MBD1 and MeCP2, and recruits H3K9me3 and H3K27me3, 5hmC progressively co-localizes with MBD3 and recruits H3K4me2 (Chen et al., 2014). Furthermore, a positive correlation has been reported between 5hmC levels and human cerebellum development (Wang et al., 2012) and 5hmC has been reported to regulate transcriptional factors involved in neurodevelopment (Szulwach et al., 2011b). Finally, alterations of 5hmC have been implicated in a number of neurodevelopmental diseases, including Rett syndrome, autism spectrum disorders, schizophrenia and fetal alcohol syndrome (Cheng et al., 2014).

Such growing evidence clearly indicates that 5hmC has an important role to play in normal neurodevelopment and maintenance of adult CNS function. Thus, it is intuitive that abnormalities of 5hmC distribution or function may also be important factors for neurodegenerative diseases. Indeed, a genome-wide study of 5hmC distribution in mouse cerebellum has revealed an age-related gene expression level-dependent enrichment of 5hmC in specific gene bodies that are linked to neurodegenerative diseases in mice and humans, including ataxia and disorders of Purkinje cell degeneration (Song et al., 2011b). The following sections describe the specific 5hmC studies that have been performed in relation to individual neurodegenerative diseases.

## AD

Alzheimer's disease (AD) is the most common neurodegenerative disorder, characterized by progressive decline of cognitive functions, neuronal cell loss, and two hallmarks of pathology, extracellular amyloid beta plaques and intracellular neurofibrillary tangles composed of hyperphosphorylated tau protein (Tanzi, 2012). The causes of AD are unknown, but some evidence has been gained from identifying abnormalities in selected genes, including the beta-amyloid precursor protein gene, *APP*, and the presenilin genes, *PSEN1* and *PSEN2*. In addition, it has been suggested that there may be alterations of epigenetic factors due to aging or in response to environmental stresses (Wang et al., 2008; Coppieters and Dragunow, 2011; Bihaqi et al., 2012). Several studies have recently investigated the global levels of DNA methylation-based enzymes and chromatin marks, DNMT1, TET1, 5mC, 5hmC, 5fC, and 5caC, in AD brain tissues using a variety of immunohistochemical detection methods, with somewhat contradictory results (Table 1). Initial studies of human brain samples, specifically the entorhinal cortex layer II of the medial temporal lobe, revealed evidence of decreased levels of 5mC and DNMT1 in neurons of AD patients (Mastroeni et al., 2010). Similar decreases in 5mC, together with decreased levels of 5hmC, were subsequently identified in the hippocampal region of AD brains (Chouliaras et al., 2013). However, further studies have shown exactly the opposite effects, reporting increased levels of both 5mC and 5hmC in AD brains (Table 1). Firstly, increased levels of 5mC have been detected in frontal cortex of AD patients (Rao et al., 2012). Secondly, increased levels of TET1, 5mC, and 5hmC, accompanied by decreases in the levels of 5fC and 5caC, have been detected in the hippocampus of AD patients, while no changes were detected in cerebellum tissues (Bradley-Whitman and Lovell, 2013). Finally, increased levels of both 5mC and 5hmC have been detected in the frontal and temporal cortex of AD patients (Coppieters et al., 2014). The reasons for the discrepancies between the different studies will require further investigation, but they may be due to the analysis of different regions of the brain and the use of different immunohistochemical quantification techniques. It will also be interesting to investigate levels of locus-specific 5hmC at specific genes related to AD pathology, such as the *APP* and *PSEN1* genes, which have previously shown AD-related changes in 5mC levels (Rogaev et al., 1994; Tohgi et al., 1999; Barrachina and Ferrer, 2009). Only after such studies will the role of 5hmC in either the causes or consequences of AD pathogenesis become apparent.

**Table 1:** **Alterations of 5hmC in neurodegenerative diseases**.

**Disease**	**5hmC effect**	**Other epigenetic effects**	**References**
AD	Not tested	Decreased global DNMT1 and 5mC in AD temporal cortex	Mastroeni et al., 2010
	Decreased global 5hmC in AD hippocampus	Decreased global 5mC in AD hippocampus	Chouliaras et al., 2013
	Not tested	Increased global 5mC in AD frontal cortex	Rao et al., 2012
	Increased global 5hmC in AD hippocampus, but no change in cerebellum	Increased global Tet1 and 5mC, and decreased global 5fC and 5caC in AD hippocampus, but no change in cerebellum	Bradley-Whitman and Lovell, 2013
	Increased global 5hmC in AD frontal and temporal cortex	Increased global 5mC in AD frontal and temporal cortex	Coppieters et al., 2014
ALS	Increased global 5hmC in sporadic ALS spinal cord	Increased global 5mC in sporadic ALS spinal cord	Figueroa-Romero et al., 2012
FXTAS	Decreased global 5hmC in rCGG FXTAS mouse model cerebellum		Yao et al., 2014
FRDA	Increased 5hmC at the 5' GAA repeat region of the *FXN* gene in FRDA cerebellum and heart	Increased 5mC at the 5' GAA repeat region and decreased 5mC at the 3' GAA repeat region of the *FXN* gene in FRDA cerebellum and heart	Al-Mahdawi et al., 2008, 2013
HD	Decreased 5hmC in the *ADORA2A* gene in HD putamen	Increased 5mC in the *ADORA2A* gene in HD putamen	Villar-Menendez et al., 2013
	Decreased global 5hmC in YAC128 HD mouse model striatum and cortex		Wang et al., 2013
PD	No change in 5hmC in 6-OHDA PD rat model striatum		Zhang et al., 2013

## ALS

Amyotrophic lateral sclerosis (ALS) is a progressive neurodegenerative disease characterized by selective loss of motor neurons within the brain and spinal cord (Calvo et al., 2014). The cause of ALS is largely unknown, although defective genes have been identified in a small percentage of familial ALS cases, including those encoding superoxide dismutase 1 (SOD1), TAR DNA-Binding Protein (TARDBP), fused in sarcoma (FUS), Ubiquilin2 (UBQLN2) and C9ORF72 (Chen et al., 2013). For the much larger percentage of sporadic ALS cases, it has been suggested that environmental factors, such as exposure to toxins or dietary factors, may be driving more global epigenetic changes (Ahmed and Wicklund, 2011). Therefore, it is interesting to note that one recent study has reported global increases in both 5mC and 5hmC levels in postmortem sporadic ALS spinal cord, but not in blood samples (Figueroa-Romero et al., 2012) (Table 1). The differences between spinal cord and blood samples suggest that neither 5mC nor 5hmC would currently be suitable as biomarkers of ALS, but further studies will no doubt follow to shed further light onto the role of 5hmC in ALS.

## FXTAS

Fragile X-associated tremor/ataxia syndrome (FXTAS) is a late-onset neurodegenerative disease caused by CGG repeat expansion mutations within the 5' untranslated region (UTR) of the *FMR1* gene (Hagerman et al., 2001). FXTAS patients carry 55–200 CGG repeats, regarded as premutation alleles, and although they do not exhibit signs of disease in early life, they generally develop severe tremor, ataxia and progressive cognitive decline in the fifth decades of life. In contrast, individuals who carry over 200 CGG repeats, regarded as full mutations, develop FXS, which is the commonest form of inherited mental retardation (Bagni and Oostra, 2013). In FXS, the CGG repeat expansion mutation becomes hyermethylated, as does the CpG island within the *FMR1* promoter region, resulting in reduced expression of *FMR1* (Naumann et al., 2009). However, in FXTAS there is increased expression of *FMR1*, and a toxic RNA gain of function is considered to be the primary disease mechanism (Jacquemont et al., 2003; Jin et al., 2003). To the best of our knowledge, there have not yet been any reports describing the levels of 5hmC at the *FMR1* locus in either FXS or FXTAS. However, one study has investigated global levels of 5hmC in the rCGG mouse model of FXTAS, which is characterized by overexpression of human CGG repeats within the 5 UTR of the *FMR1* gene in Purkinje neuronal cells, leading to cell death and subsequent behavioral deficits (Hashem et al., 2009). The results showed there to be a genome-wide decrease in 5hmC levels in the cerebellum tissues of rCGG mice compared with age-matched wild-type controls, mainly within gene bodies and CGIs (Yao et al., 2014). However, there were also increases of 5hmC levels in repetitive elements and cerebellum-specific enhancers that correlated with genes and transcription factors known to be involved in neurodevelopment (Table 1). These findings strongly suggest a potential function for 5hmC as part of the disease mechanism for FXTAS.

## FRDA

Friedreich ataxia (FRDA) is a rare autosomal recessive neurodegenerative disorder caused by GAA repeat expansion mutation within intron 1 of the *FXN* gene, leading to decreased expression of the essential mitochondrial protein frataxin (Campuzano et al., 1996). The main sites of pathology are the large sensory neurons of the dorsal root ganglia and the dentate nucleus of the cerebellum (Koeppen, 2013), although non-CNS pathologies are also evident, including hypertrophic cardiomyopathy (Weidemann et al., 2013) and diabetes (Cnop et al., 2013). Several studies have identified FRDA-related epigenetic changes, including alterations of DNA methylation status, in the immediate vicinity of the expanded GAA repeats of the *FXN* gene (Evans-Galea et al., 2013; Sandi et al., 2014). An initial investigation of DNA methylation revealed hypermethylation of specific CpG sites upstream of the GAA repeat in FRDA patient lymphoblastoid cells compared to unaffected controls (Greene et al., 2007). Our group subsequently identified hypermethylation at the upstream GAA repeat region in FRDA postmortem cerebellum and heart tissues (Al-Mahdawi et al., 2008). Interestingly, we also identified reduced hypomethylation in the downstream GAA repeat region in FRDA patient tissues compared with controls. These findings were confirmed in blood and buccal cell samples from a large cohort of FRDA patients, where a significant inverse correlation was also detected between the level of DNA methylation in the upstream GAA region and the level of *FXN* expression (Evans-Galea et al., 2012). Yet another study has shown that the degree of DNA methylation in the upstream GAA repeat region in FRDA patients correlates with the length of the GAA repeats and inversely correlates with the age of disease onset (Castaldo et al., 2008). Therefore, there is good evidence that DNA methylation may have an, as yet unknown, role to play in the molecular mechanism of FRDA. With this in mind, our group have recently analyzed the 5hmC status of one of the *FXN* upstream GAA CpG sites in FRDA cerebellum and heart tissues using a restriction enzyme-based procedure that allowed distinction between 5hmC and 5mC and we found that the majority of the hypermethylated DNA at this CpG residue comprises 5hmC rather than 5mC (Al-Mahdawi et al., 2013) (Table 1). It is possible that raised 5hmC levels reflect an attempt to reverse GAA repeat-induced *FXN* gene silencing, which is marked by increased DNA methylation at the upstream GAA repeat region, rather than indicating any involvement in the cause of disease. Therefore, it will be interesting to see if there are further 5hmC alterations of the *FXN* gene, or indeed other genes, associated with FRDA.

## HD

Huntington's disease (HD) is an autosomal dominant progressive neurodegenerative disease, characterized by chorea, dystonia, and cognitive decline, with the main site of pathology being the striatum. HD is caused by CAG repeat expansion mutation within exon 1 of the *HTT* gene, leading to abnormal polyglutamine formation within the amino-terminus of the HTT protein (MacDonald et al., 2003). The mechanism of disease is unknown, although many studies have provided evidence for polyglutamine or RNA toxic gains of function as well as haploinsufficiency or alternative splicing of the *HTT* gene as potential causes (Landles and Bates, 2004; Sathasivam et al., 2013). More recently, evidence has been put forward from two studies to suggest the potential involvement of 5hmC in HD by two distinct mechanisms (Table 1). Firstly, increased levels of 5mC and decreased levels of 5hmC were identified in the 5' UTR of the *ADORA2A* gene in the striatum (specifically the putamen) of HD patients compared with age-matched controls (Villar-Menendez et al., 2013). The *ADORA2A* gene encodes the adenosine A_2A_ receptor, a G-protein-coupled receptor that is normally highly expressed in the basal ganglia, but severely reduced in HD (Glass et al., 2000). Secondly, genome-wide loss of 5hmC has been reported in YAC128 HD mouse striatum and cortex brain tissues compared with age-matched wild-type controls (Wang et al., 2013). A closer inspection of this data revealed 747 differentially hydroxymethylated regions in the striatum of which 49 showed HD-related increases of 5hmC, enriched in gene bodies and positively correlated with gene transcription, and 698 showed HD-related decreases of 5hmC. The authors speculate that 5hmC is a novel epigenetic feature in HD, involved in neurogenesis, neuronal function and survival in HD brain.

## PD

Parkinson's disease (PD) is the second most common neurodegenerative disease after AD, characterized by the progressive loss of substantia nigra dopaminergic neurons, resulting in muscle rigidity, bradykinesia, tremor, and instability. The causes of PD are unknown, but mutations in several genes have now been identified in rare cases of inherited PD, including the genes *SNCA* (alpha-synuclein), *PARK2* (parkin), *PTEN* induced Putative Kinase 1 (*PINK1*), *PARK7* (DJ-1), Leucine Rich Repeat Kinase 2 (*LRRK2*) and *ATP13A2* (Yang et al., 2009). Previous studies have suggested the potential involvement of DNA methylation in PD, particularly with regards to regulation of *SNCA* gene expression, but without solid conclusions (Lu et al., 2013). More recently, 5hmC levels have been studied in striatal brain tissues of the 6-OHDA induced rat model of PD, but while 5hmC content generally increased with age, no changes in 5hmC levels were detected compared with controls (Zhang et al., 2013). Therefore, any potential involvement of 5hmC in the causes or consequences of PD remain to be identified.

## Conclusions: implications for diagnosis and therapy of neurodegenerative diseases

To date, the investigations of 5hmC in relation to neurodegenerative diseases can be considered to be within their early days. Of the few studies that have been performed, no common features have emerged regarding 5hmC alterations and neurodegenerative diseases; both global increases and decreases of 5hmC levels have been identified in different diseases, and in the case of AD, within the same disease. It is likely that poorly reproducible techniques of immunohistochemical quantification have contributed to this lack of clarity. One impetus behind the recent studies of 5hmC is the potential to identify changes in 5hmC as a potential biomarker of neurodegenerative disease. Alterations of 5hmC levels may be useful, irrespective of whether they are considered to be a cause or a consequence of the disease process. However, initial studies of sporadic ALS have revealed no correlation between alterations of 5hmC, or indeed in 5mC, in spinal cord and blood, suggesting that these would not be suitable as biomarkers of this neurodegenerative disease (Figueroa-Romero et al., 2012). Another driver behind 5hmC studies of neurodegenerative disease is the potential to identify novel targets for therapy. In this case, alterations of 5hmC levels would have to be involved in the molecular disease process rather than being a consequence of disease. However, if 5hmC does indeed prove to be a significant epigenetic mark involved in the causation of neurodegenerative disease, then it may be possible to develop drugs that modify the 5hmC status, either to decrease 5hmC levels by inhibition of TET enzyme activity (Xiao et al., 2012) or to increase 5hmC levels by enhancement of TET enzyme activity (Yin et al., 2013). However, further investigations of 5hmC alterations are first required for all neurodegenerative diseases, both at global and locus-specific levels. Such studies will be enhanced by the development of state of the art technologies, such as the recently described third-generation sequencing and oxidative bisulfite (oxBs) BeadChip platforms (Song et al., 2011a; Stewart et al., 2014).

## Author contributions

All authors contributed to draft the manuscript and all authors read and approved the final manuscript.

### Conflict of interest statement

The authors declare that the research was conducted in the absence of any commercial or financial relationships that could be construed as a potential conflict of interest.
